# The Impact of Estradiol and 1,25(OH)2D3 on Metabolic Syndrome in Middle-Aged Taiwanese Males

**DOI:** 10.1371/journal.pone.0060295

**Published:** 2013-03-28

**Authors:** Kai-Hung Cheng, Shu-Pin Huang, Chun-Nung Huang, Yung-Chin Lee, Chih-Sheng Chu, Chu-Fen Chang, Wen-Ter Lai, Chia-Chu Liu

**Affiliations:** 1 Division of Cardiology, Department of Internal Medicine, Kaohsiung Medical University Hospital, Kaohsiung, Taiwan; 2 Faculty of Medicine, College of Medicine, Kaohsiung Medical University, Kaohsiung, Taiwan; 3 Graduate Institute of Medicine, College of Medicine, Kaohsiung Medical University, Kaohsiung, Taiwan; 4 Department of Urology, Kaohsiung Medical University Hospital, Kaohsiung, Taiwan; 5 Department of Urology, College of Medicine, Kaohsiung Medical University, Kaohsiung, Taiwan; 6 Cancer Center, Kaohsiung Medical University Hospital, Kaohsiung, Taiwan; 7 Department of Physical Therapy, Tzu Chi University, Hualien, Taiwan; 8 Pingtung Hospital, Department of Health, Executive Yuan, Pingtung, Taiwan; Indiana University, United States of America

## Abstract

In addition to adipocytokines, estradiol (E2) and vitamin D have been reported to affect insulin sensitivity, glucose homeostasis and body weight. However, studies about the impact of E2 and vitamin D on metabolic syndrome (MetS) are still limited. The aim of this study is to clarify the roles of circulating E2 and vitamin D on the risk of MetS in middle-aged Taiwanese males. A total of 655 male volunteers, including 243 subjects with MetS (mean age: 56.7±5.8 years) and 412 normal controls (mean age: 55.1±3.6 years), were evaluated. Subjects with MetS had significantly lower circulating E2, 1,25(OH)_2_D_3_, and adiponectin, and higher leptin than those without MetS (P<0.001 for all comparisons). E2 and 1,25(OH)_2_D_3_ were significantly associated with 4 individual components of MetS; more than adiponectin and leptin that were only associated with 3 individual components. In multivariate regression analysis, E2 (beta = −0.216, P<0.001) and 1,25(OH)_2_D_3_ (beta = 0.067, P = 0.045) were still significant predictors of MetS independent of adiponectin and leptin. Further large studies are needed to confirm our preliminary results and elucidate the possible mechanism.

## Introduction

Metabolic syndrome (MetS) is a collection of cardiometabolic risk factors, including obesity, insulin resistance, hypertension, and dyslipidemia. Both MetS and type 2 diabetes mellitus (T2DM) are closely related to the increasing risk of developing coronary heart disease and cardiovascular disease (CVD) [1], [2], [3], [4], [5], [6], [7] which could manifest severe and fatal consequences. Therefore, the prevention of MetS is essential.

Numerous factors, such as hormones, adipocytokines and vitamin D have been reported to be associated with the pathogenesis of MetS. Estradiol (E2) has been reported to be able to affect insulin sensitivity, glucose homeostasis, body weight and adiposity [8], [9]. In addition, adipocytokines like adiponectin and leptin are known to play important roles in the development of MetS. Low adiponectin status has been reported to be associated with obesity, MetS, and CVD [10], [11], [12], [13], while high leptin or leptin resistance status has been reported to be associated with obesity, MetS and CVD [13], [14], [15], [16].

The best model proposed, which links sex hormones with MetS, is polycystic ovary syndrome(POS). POS, a common disorder for women, is associated with a low estradiol-to-testosterone ratio in oligo-anovulatory cycles and atherogenic lipidic patterns resulting from the suppression of E2 production by potent endogenous aromatase inhibitors [17], [18], [19]. Recently, Saltiki et al reported that endogenous estrogen levels were related to endothelial function in males independent of lipid levels [20]. However, association studies between E2 and MetS in the middle-aged male population are still limited.

Vitamin D deficiency has been reported to be associated with increased risk of CVD, including hypertension, heart failure, and ischemic heart disease [21] in addition to MetS [22], [23], [24], [25]. A significant, positive correlation between 25-hydroxyvitamin D (25(OH)D) levels and adiponectin levels was also found in subjects with abnormal glucose tolerance and in a young Middle-Eastern population [26], [27]. 1alpha-hydroxylase, responsible for the final step in vitamin D activation, is associated with type 1 DM and is responsible for calcitriol-related complications and MetS [28], [29], [30], [31]. In contrast to 25(OH)D, calcitriol (the activated form of vitamin D also known as 1,25-dihydroxyvitamin D or 1,25(OH)_2_D_3_) works on cardiac muscle directly, regulates hormone secretion of the parathyroid gland, and affects the renin-angiotensin-aldosterone system, and immune system [32]. However, the associations between vitamin D (1,25(OH)_2_D_3_ or calcitriol) levels and the risk of Met or its individual components have not yet been completely clarified.

Therefore, the aim of this study was to evaluate the impact of the levels of E2 and 1,25(OH)_2_D_3_, beyond adipocytokines, on the risk of MetS and its individual components in a middle-aged Taiwanese male population.

## Materials and Methods

### Subjects and study protocol

The cross-sectional data of 694 Taiwanese males (age: 44–77 years) were collected from a free health screening held by a medical center in Kaohsiung city, Taiwan. Ethics approval following the Declaration of Helsinki was authorized by the Institutional Research Ethics Committee of Kaohsiung Medical University Hospital and informed written consent was obtained from each participant. Men who had previously been diagnosed with hypertension, DM, or hyperlipidemia (kept under control by regular medication) were included in the study, but men who were diagnosed as labile for hypertension, labile for diabetes, having current malignancy, advanced liver and/or renal disease or who were using hormones, antiandrogen treatment, antifungal drugs, or steroidal agents were excluded [3], [33].

A complete medical, surgical, and psychosexual history and the results from detailed physical examinations, including measurement of the body weight, height, and blood pressure, were recorded for each subject. Fasting blood samples were also taken for further biochemical analysis and hormone profiling. Body mass index (BMI) (kg/m^2^) was calculated as the ratio of the body weight and the square of body height. Subjects were classified as alcohol drinkers, cigarette smokers, or betel nut chewers if they had regularly consumed any alcoholic beverage ≥1 times per week, had smoked ≥10 cigarettes per week, or had chewed ≥7 betel quids per week respectively, for at least 6 months. Current users were those who were still using any of these substances within one year before the interview. Former users were defined as those who had stopped any of these habits for at least 1 year before interview [34], [35]. Hypertension was defined by a systolic blood pressure (SBP) of ≥140 mmHg or a diastolic blood pressure (DBP) of ≥90 mmHg, while hyperlipidemia was defined by a total cholesterol level of ≥200 mg/dL or a triglycerides level of ≥200 mg/dL [3], [36]. DM was diagnosed when the fasting blood glucose (FBG) was ≥126 mg/dL. An individual was diagnosed with MetS if he was positive for at least three of the five following criteria: (1) waist circumference (WC) ≥90 cm; (2) high density lipoprotein (HDL) cholesterol <40 mg/dL; (3) triglyceride (TG) ≥150 mg/dL; (4) blood pressure (BP) ≥130/85 mm Hg or diagnosed as hypertensive and on therapy; (5) fasting blood glucose (FBG) ≥100 mg/dL or diagnosed as type 2 diabetes mellitus (DM), in accordance with the modified criteria proposed by the Bureau of Health Promotion in Taiwan [3], [7], [37].

### Biochemical analysis using radioimmunoassay

Peripheral venous blood samples after more than 8 hours fasting overnight were drawn into pyrogen-free tubes for analysis of serum glucose, lipid panels, and routine biochemical profiles. Through radioimmunoassay (RIA), E2 was measured using the Siemens' RIA kits (Los Angeles, USA; Inter-assay coefficient of variation (CV): 4.0%∼7.0%; Inter-assay CV: 4.2%∼8.1%), while 1,25(OH)_2_D_3_ was determined using the DiaSorin RIA kits (Northwestern Avenue Stillwater, USA; Intra-assay CV: 6.8%∼11.3%; Inter-assay CV:12.3%∼15.3%). Leptin and adiponectin were measured using Millipore's RIA kits (Missouri, USA; Intra-assay CV: 3.4%∼8.3% and 1.78%∼6.21% respectively; Inter-assay CV: 3.0%∼6.2% and 6.90%∼9.25% respectively). SHBG levels (detectable range: 0.2–180 nmol/L; inter-assay CV of 4.8%, and intra-assay CV of 3.5%) were determined using a DPC Immulite analyzer (Diamond Diagnostics, Holliston, MA). Free E2 was calculated based on mass action laws with Vermeulen's formula [38], [39].

### Statistical analysis

Quantitative demographic and laboratory data were presented as mean ± standard deviation (SD). To quantify the differences between subjects with MetS and without MetS, qualitative variables were compared using the chi-square test and Fisher's exact test, while quantitative variables were compared using the Student's *t*-test. A one-way analysis of variance (ANOVA) with the LSD post-hoc test was used to compare the differences in the quantitative variables between the various components of MetS. Correlations between clinical characteristics, biochemical variables, and MetS were analyzed by Spearman's correlation with a correlation coefficient (r) of 0–0.25 indicating little to no correlation, 0.26–0.50 for a fair correlation, 0.51–0.75 for a moderate to good correlation, greater than 0.76–0.99 for a good to excellent correlation, and 1.00 for a perfect linear relationship. Any variables with significant association with the risk of MetS in the initial analyses were further examined in multivariate regression analyses to determine the independent risk factors for MetS. SPSS version 18.0 (SPSS Inc., Chicago, IL, USA) was used for all statistical analyses.

## Results

### Baseline characteristics

Among the 694 participants, 39 were excluded from the study because they failed to follow through with the complete biochemistry profile, thus, 655 men were included in this study. Participants were divided into two groups according to the presence or absence of MetS. The baseline characteristics and biochemical data of the subjects with MetS (n = 243) and without MetS (n = 412) are summarized in **Table 1** **.** Subjects with MetS had significant increases in age, BMI, current habits of smoking, drinking, betel quid chewing, and prevalence of CVD when compared to those without MetS. The MetS group also had significantly higher level of leptin, but lower levels of adiponectin, E2, and 1,25(OH)_2_D_3_. However, free E2 level was not significantly different between the two groups.

**Table 1:** Means ± standard deviations of the baseline characteristics and biochemical variables in the subjects with and without MetS.

	Subjects without MetS (n = 412)	Subjects with MetS (n = 243)	*P* value
Age (yrs)	55.1±3.6	56.7±5.8	<0.001*
BMI (Kg/m^2^)	24.4±2.3	27.6±9.1	<0.001*
Waist circumference (cm)	83.4±5.6	90.6±6.8	<0.001*
Hip circumference (cm)	95.3±6.7	106.8±86.8	0.039*
Waist-hip ratio	0.96±1.83	0.91±0.08	0.21
SBP (mm-Hg)	128.4±11.8	136.5±10.8	<0.001*
DBP (mm-Hg)	80.5±8.2	86.3±8.5	<0.001*
DM n (%)	16 (3.9)	47 (19.3)	<0.001*
Hypertension, n (%)	71 (17.2)	115 (47.3)	<0.001*
Dyslipidemia history, n (%)	40 (9.7)	89 (36.6)	<0.001*
CVD history, n (%)	16 (3.9)	29 (11.9)	<0.001*
Smoking, n (%)			0.002*
Nonsmokers	315 (76.5)	160 (65.8)	
Former Smokers	57 (13.8)	37 (15.2)	
Current Smokers	40 (9.7)	44 (18.1)	
Drinking, n (%)			0.002*
Non-drinker	356 (86.4)	182(74.9)	
Former Drinkers	6 (1.5)	11 (4.5)	
Current Drinkers	50 (12.1)	50 (20.6)	
Betel quid, n (%)			0.022*
Never Chewed	404 (98.1)	227 (93.4)	
Former Chewers	6 (1.5)	13 (5.3)	
Current Chewers	1 (0.2)	2 (0.8)	
Blood Biochemistry			
Fasting glucose (mg/dl)	94.0±14.1	109.1±24.8	<0.001*
TG (mg/L)	108.6±85.0	177.6±92.7	<0.001*
Total cholesterol (mg/dl)	189.5±33.9	190.3±31.6	0.77
HDL-C (mg/dl)	51.6±11.0	41.8±8.5	<0.001*
Adiponectin (ng/ml)	14.3±6.9	7.8±4.4	<0.001*
Leptin (ng/ml)	3.3±1.9	5.3±2.7	<0.001*
1,25(OH)_2_D_3_ (pg/ml)	47.7±19.2	40.6±16.1	<0.001*
E2 (pg/ml)	26.3±8.1	19.7±9.1	<0.001*
Free E2 (pg/ml)	0.7±0.3	3.0±40.0	0.35
SHBG(nmol/L)	49.7±24.7	37.0±15.5	<0.001*
Albumin(g/dl)	4.4±0.2	4.5±0.2	<0.001*

BMI: body mass index; SBP: systolic blood pressure; DBP: diastolic blood pressure; DM: diabetes mellitus; CVD: cardiovascular disease; TG: triglyceride; HDL: high density lipoprotein; CHOL(T): total cholesterol; E2: estradiol; SHBG: sex hormone–binding globulin;

* : Significant difference (P<0.05).

### Clinical characteristics and biochemical variables in subjects with various number of MetS components

**Figure 1:**
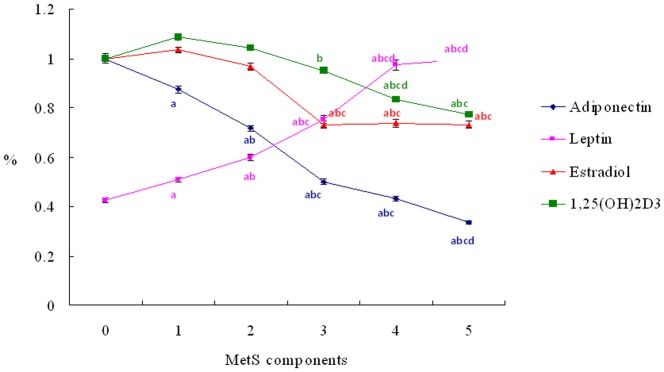
Ensemble-averaged levels ± standard error of the adiponectin, leptin, E2, and 1,25(OH)_2_D_3_ as the numbers of the MetS components increased. The reference points of “100 %” for the adiponectin, leptin, E2 and 1,25(OH)_2_D_3_ are 16.77 ng/ml, 6.28 ng/ml, 25.99 pg/ml and 45.36 pg/ml respectively. ^a^ P<0.05 as compared to the subjects without MetS components; ^b^ P<0.05 as compared to subjects with one of the MetS components; ^c^ P<0.05 as compared to subjects with two of the MetS components; ^d^ P<0.05 as compared to subjects with three of the MetS components; ^e^ P<0.05 as compared to subjects with four of the MetS components.

The clinical characteristics and biochemical data of subjects with various numbers of MetS components are summarized in **Table 2**. As the number of MetS components increased, age, BMI, and leptin level increased, but adiponectin, E2, and 1,25(OH)_2_D_3_ levels decreased significantly (P<0.001 for all comparisons). Furthermore, as the number of MetS components increased, adiponectin and 1,25(OH)_2_D_3_ decreased but leptin levels increased in a linear shape. Only E2 levels decreased in a ladder shape as the numbers of MetS components increased. (**Figure 1**)

**Table 2:** Means ± standard deviations of the clinical characteristics and biochemical variables in subjects with various numbers of metabolic syndrome (MetS) components.

	Subjects without MetS			Subjects with MetS			*P* value
Numbers of MetS Components	0 (n = 95)	1 (n = 183)	2 (n = 134)	3 (n = 139)	4 (n = 82)	5 (n = 22)	
Age (yrs)	54.8±3.0	55.1±3.0	55.3±4.6	56.3±5.2^a^ ^b^	57.3±6.7^a^ ^b^ ^c^	57.3±6.2^a^ ^b^	<0.001*
BMI (Kg/m^2^)	23.3±2.1	24.3±2.1	25.1±2.4^a^	26.2±2.3^a^ ^b^	29.6±15.1^a^ ^b^ ^c^	28.9±2.3^a^ ^b^ ^c^	<0.001*
Adiponectin (ng/ml)	16.8±8.3	14.7±6.6^a^	12.0±5.5^a^ ^b^	8.4±4.6^a^ ^b^ ^c^	7.2±4.2^a^ ^b^ ^c^	5.6±3.1^a^ ^b^ ^c^ ^d^	<0.001*
Leptin (ng/ml)	2.7±1.4	3.2±1.6^a^	3.8±2.3^a^ ^b^	4.7±2.3^a^ ^b^ ^c^	6.1±3.2^a^ ^b^ ^c^ ^d^	6.3±1.9^a^ ^b^ ^c^ ^d^	<0.001*
E2 (pg/ml)	26.0±8.4	26.9±7.6	26.0±8.8	19.6±9.1^a^ ^b^ ^c^	19.8±9.4^a^ ^b^ ^c^	19.7±10.0^a^ ^b^ ^c^	<0.001*
1,25(OH)_2_D_3_ (pg/ml)	45.4±16.2	49.3±21.5	47.3±18.0	43.1±16.3^b^	37.8±15.4^a^ ^b^ ^c^ ^d^	35.1±15.8^a^ ^b^ ^c^	<0.001*

BMI: body mass index; E2: estradiol;

* : Significant difference (P<0.05);

a P<0.05 as compared to the subjects without MetS components;

b P<0.05 as compared to subjects with one of the MetS components;

c P<0.05 as compared to subjects with two of the MetS components;

d P<0.05 as compared to subjects with three of the MetS components;

^e^ P<0.05 as compared to subjects with four of the MetS components.

### Correlations between biochemical variables and individual MetS components

Adiponectin was correlated with WC (r = 0.57, P<0.001), HDL (r = 0.46, P<0.001) and TG (r = −0.30, P<0.001). Leptin was correlated with HDL (r = −0.27, P<0.001), WC (r = 0.63, P<0.001), and DBP (r = 0.28, P<0.001); E2 level was correlated with WC (r = −0.13, P = 0.001), FBG (r = −0.16, P<0.001), TG (r = −0.11, P = 0.006), and HDL (r = −0.20, P<0.001). 1,25(OH)_2_D_3_ was correlated with WC (r = −0.12, P = 0.003), FBG (r = −0.13, P = 0.001), TG(r = −0.09, P = 0.02), and HDL(r = −0.11, P = 0.007). In total, adiponectin and leptin were significantly associated with 3 MetS components, but E2 and 1,25(OH)_2_D_3_ were significantly associated with 4 MetS components.

### Multivariate regression analyses

All of the clinical and biochemical parameters which were not diagnostic parameters for MetS and were significantly different between subjects with and without MetS, were placed into a multivariate regression model. In model 1 with adjustment for age, BMI, and personal habits (smoking, alcohol drinking and betel quid chewing), adiponectin (beta = −0.421, P<0.001), leptin (beta = 0.111, P = 0.002), E2 (beta = −0.321, P<0.001) and 1,25(OH)_2_D_3_ (beta = −0.153, P<0.001) were all significantly independent predictors of MetS (**Table 3**). In full adjusted model 2 after considering age, BMI, personal habits (smoking, alcohol drinking, and betel quid chewing), SHBG and above 4 factors (adiponectin, leptin, E2 and 1,25(OH)2D3), E2 (beta = −0.216, P<0.001) and 1,25(OH)2D3(beta = 0.067, P = 0.045) were still indepndent predictors of MetS, in addition to adiponectin (beta = −0.259, P<0.001) and leptin (beta = 0.086, P = 0.007) (**Table 3**).

**Table 3:** Multivariate regression analyses for the associations of circulating adiponectin, E2, leptin, 1,25(OH)_2_D_3_ levels and metabolic syndrome (MetS).

Variables	β (standardized coefficient)	SE	t	95% Confidence Interval (CI)	P-value
Model 1: adjustment for age, BMI, and personal habits (smoking, alcohol drinking and betel quid chewing)					
Adiponectin	−0.421	0.002	−12.510	(−0.034∼−0.025)	<0.001*
E2	−0.321	0.002	−9.243	(−0.021∼−0.014)	<0.001*
Leptin	0.111	0.001	3.069	(0.001∼0.006)	0.002*
1,25(OH)_2_D_3_	−0.153	0.001	−4.172	(−0.006∼0.002)	<0.001*
(B) Model 2: adjustment for age, BMI, personal habits (smoking, alcohol drinking and betel quid chewing), SHBG and all of above 4 factors (adiponectin, E2, leptin, and 1,25(OH)_2_D_3_ levels)( R^2^ = 0.438).					
Adiponectin	−0.259	0.003	−7.054	(−0.023∼−0.013)	<0.001*
E2	−0.216	0.002	−6.397	(−0.015∼−0.008)	<0.001*
Leptin	0.086	0.001	2.335	(0.001∼0.005)	0.007*
1,25(OH)_2_D_3_	−0.067	0.001	−2.010	(−0.003∼0.000)	0.045

BMI: body mass index; E2: estradiol; SHBG: sex hormone–binding globulin;

* : Significant difference (P<0.05).

## Discussion

In addition to adiponectin and leptin, this cross-sectional study found that circulating E2 and vitamin D3 levels were significantly associated with the risk of MetS and its individual components in middle-aged Taiwanese males. Subjects with MetS were found to have significantly lower circulating E2, 1,25(OH)_2_D_3_, and adiponectin levels as well as significantly higher leptin levels when compared to those without MetS (Table 1). With increasing numbers of MetS components, there were linear-shaped declines in 1,25(OH)_2_D_3_ and adiponectin; linear-shaped increases in leptin levels; and a ladder-shaped decline in E2 levels (Figure 1). In addition, correlation analyses also showed that E2 and 1,25(OH)_2_D_3_ were significantly associated with 4 individual components of MetS; more than adiponectin and leptin that were only associated with 3 individual components of MetS. The multivariate regression analyses also confirmed that E2 and 1,25(OH)_2_D_3_ were significant predictors of MetS independent of adiponectin and leptin (Table 3).

Previous studies have found that low E2 was associated with obesity and MetS in productive females with PCO, and adult males with the aromatase gene mutation. [17], [18], [19], [29], [31], [40], [41], [42], [43]. In our study, we also found that low E2 was significantly associated with MetS in middle-aged males. This is in contrast to findings reported by Maggio et al that found an independent association of increased E2 with MetS in an elderly male population [44]. Therefore, E2 might have contrary influences on MetS in middle-aged and elderly male populations. The result of low E2 with MetS in our study is consistent with the low estradiol-to-testosterone ratio seen in polycystic ovary syndrome with MetS, which is also associated with oligo-anovulatory cycles, atherogenic lipidic pattern, and insulin resistance [17], [18], [19], [45]. E2 and its receptor play important physiological and protective roles in the reproductive ages of both males and females. For males, E2 acts to prevent apoptosis of sperm cells [46] and works in vascular protection and modulation of inflammation [47], [48]. However, hormone replacement therapy (HRT) during menopause has turned out to be a harmful risk factor in that it increases the risk of stroke, venous thromboembolism, and coronary heart disease [49], [50]. This may be associated with the age-related decrease in estrogen receptor-mediated vascular relaxation [51] and may be used to elucidate the protective effects of E2 at reproductive ages and the contrary influences of increased E2 for the elderly or menopausal populations. Furthermore, E2 is also known to modulate insulin sensitivity and glucose homeostasis, and E2 supplementation has been shown to aid mice treated with a high fat diet in order to overcome central leptin resistance [8], [9], [52].

In addition, our study is the first to report an association between 1,25(OH)_2_D_3_ levels and the risk of MetS. Previous studies had reported that low 25(OH)D levels were significantly associated with insulin resistance and an increased risk of MetS [22], [23], [24], [26], [53], [54], [55], and was considered to be a risk factor for CVD [21]. Recently, 1,25(OH)_2_D_3_, the activated form of vitamin D, was suggested to play important roles in the insulin sensitivity and glucose metabolism [56] and was able to improve the weight-related inflammation and insulin resistence [57]. Studies on clinial intervention also found that vitamin D3 supplement can reduce the body fat, especially the visceral fat and lipid metabolism in subjects with obesity [58], [59], [60], [61], which may further decrease the risk of MetS. In our study, we found that 1,25(OH)_2_D_3_ levels displayed a linear decline as the numbers of MetS components increased in middle-aged males, which supported that a reduction in 1,25(OH)_2_D_3_ levels may be a contributing factor to an increased risk of developing MetS. However, an alternative explanation is that obesity and MetS can increase the inflmmation status [62], [63], which may further influence the level of 1,25(OH)_2_D_3_. Further studies are needed to evalute the real causal-relationships among 1,25(OH)_2_D_3_, inflammation and MetS.

In our study, we also found that adiponectin level was negatively correlated with the number of MetS components. Recently, Zhuo et al also reported that adiponectin concentrations decreased with increasing MetS components in older Chinese adults [64], which is consistent with our findings. HDL has been found to affect adipocyte metabolism and adiponectin expression [65]. Our study also found that adiponectin level was significantly corrected with HDL in our study population. In addition, previous studies also indicate that adiponectin and E2 are interactive. Adiponectin increases insulin-like growth factor I-induced estradiol secretion [66], influences adjacent epithelial function by estrogen receptor(ER) -dependent and ER-independent mechanisms to reduce breast cancer risk [67] and E2 suppresses the adiponectin-regulated OPG/RANKL expression to inhibit osteoclastogenesis-related inflammation and bone resorption [68]. Therefore, low adiponectin in MetS may disturb its regulation in E2 and increase the associated inflammation, which may also lead to the decrease of circulating E2 levels. Further studies are needed to evalute the real causal-relationships among adioponetin, E2 and MetS.

Our study also found that leptin level was positively correlated with the number of MetS components. Previous studies also reported that high leptin or leptin resistance status was associated with obesity, MetS and CVD [13], [14], [15], [16] , which is consistent with our results. In addition to smoking and alcohol drinking, our study also found that subjects with MetS had significantly higher prevalence of betel quid chewing than those without MetS. Arecoline, a major alkaloid in betel nuts, has been reported to have an impact on adipogenic differentiation (adipogenesis), lipolysis, and glucose uptake by fat cells [69] and contributes to the formation of MetS [70] and CAD [71]. Further large studies may be needed to elucidate the possible mechanisms of betel quid chewing on the risk of MetS.

There are some limitations in this study. First, our data was based on community-dwelling men participating in a free health screening. Although the screening was open to the general male population, some selection bias may have existed. Other large population-based studies may be needed to confirm our preliminary results. Second, this is a cross-sectional study that can only evaluate the possible associations among those factors, MetS and its individual components. However, we can not evaluate the real causal-relationships. Further prospective studies are needed to elucidate the possible effects of those factors on the risk of MetS and its individual components. Third, we did not evaluate the effect of total and free testosterone levels which may play important roles in the risk of MetS and circulating E2 levels via aromatization.

## Conclusions

In addition to adiponectin and leptin, E2 and 1,25(OH)_2_D_3_ were significantly associated with the risk of MetS and its individual components in middle-aged males. With increasing numbers of MetS components, there were a linear-shaped decline in 1,25(OH)_2_D_3_ and a ladder-shaped decline in E2 levels. Further large studies are needed to confirm our preliminary results and elucidate the possible mechanism.
